# CircSMOX in muscle and neuronal physiology and disease

**DOI:** 10.1007/s00726-026-03549-7

**Published:** 2026-09-18

**Authors:** Marianna Nicoletta Rossi, Lavinia Attili, Manuela Cervelli

**Affiliations:** 1 Department of Sciences, University of Roma Tre, 00146 Rome, Italy; 2 LIFE Institute for Research and Health Care, Santa Lucia IRCCS, 00179 Rome, Italy

**Keywords:** CircRNA, NcRNA, CircSMOX, Neural tissue, Skeletal muscle tissue

## Abstract

Circular RNAs (circRNAs) are single stranded RNA molecules with a covalently closed loop structure that confers remarkable stability. They are increasingly recognized as important regulators of gene expression, showing tissue- and stage-specific expression patterns. CircRNAs influence gene expression through several routes: many sequester microRNAs, others bind specific RNA-binding proteins, and some affect transcription or post-transcriptional control. A subset can even be translated into short peptides. They are particularly abundant in the nervous system and in skeletal muscle, where they contribute to processes such as neural development, synaptic function, neuronal plasticity, muscle formation, and tissue homeostasis. Disruptions in circRNA expression patterns have been linked to neurodegenerative conditions, muscle-wasting disorders, and other disease states. Among the known circRNAs, circSMOX-formed from exons 2 and 3 of the murine *SMOX* gene-has been found dynamically expressed during brain and muscle development and increased in models of ischemia, spinal cord damage, and muscle atrophy. During C2C12 myogenic differentiation, circSMOX expression parallels that of the linear SMOX transcript. In contrast, in dexamethasone-induced atrophy and in ALS mouse models (SOD1^G93A^ and hFUS^+/+^), which exhibit progressive muscle atrophy, circSMOX is significantly upregulated. Mainly localized in the cytoplasm and less abundant than the linear isoform, circSMOX acts as a miR-340-5p sponge, influencing neuroinflammatory pathways. Despite these insights, the precise molecular mechanisms of circSMOX remain to be fully clarified, and the identification of a human ortholog will be important to broaden its therapeutic potential.

## circRNA biogenesis and functions

**Fig. 1:**
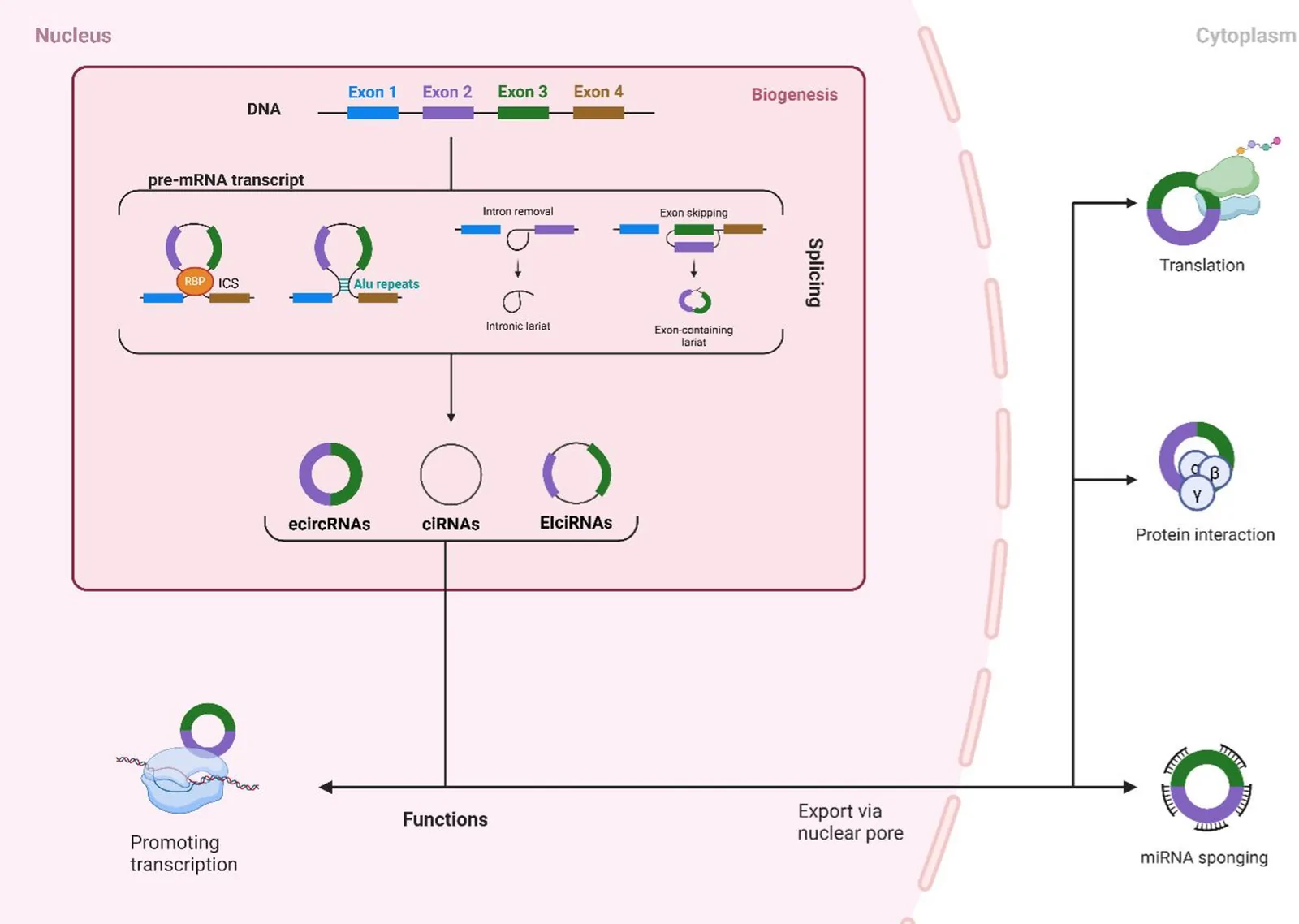
Biogenesis and functions of circular RNA. Circular RNAs can arise from pre-mRNA transcript by: intron pairing facilitated by RNA binding proteins (RNAB) and/or by Alu repeats pairing, by intron-lariat formation and by exon-skipping and lariat formation. Three types of circRNA can be produced, ecircRNA, ciRNA and EIciRNAs. circRNAs can function in the nucleus (promoting transcription) or in the cytoplasm, where they can be translated or they can interact with proteins (protein interaction) or with miRNA (miRNA sponging)

Circular RNAs (circRNAs) were firstly identified in eucaryotic cells in 1979 (Hsu and Coca-Prados 1979), but their functional roles and importance in regulating the homeostasis of cells and tissues remained unexplored for a long time. In the last 15 years, circRNA have become key factors in the regulation of gene expression and circular products arising from thousands of human and mouse genes have already been described (Misir et al. 2022). Growing body of evidence demonstrated that circRNAs expression is developmentally regulated and tissue and cell-type specific (Rybak-Wolf et al. 2015). The majority of circRNA arise from precursor mRNAs transcribed by RNA polymerase II, through a process in which a downstream 3′ splice site is covalently joined to an upstream 5′ splice site, generating a closed-loop structure via back-splicing (Chen 2020). Another route of circRNA formation is through intron lariats that fail to be debranched (Zhang et al. 2013). The back-splicing activity is catalysed by the spliceosomal machinery and is regulated by intronic complementary sequences (ICSs) in flanking introns of circle-forming exons (Jeck et al. 2013). In humans, ICSs are often Alu elements that pair to promote back-splicing (Ebbesen et al. 2017; Misir et al. 2022). Several RNA Binding Proteins (RBPs) positively or negatively regulate circRNA biogenesis, both facilitating or inhibiting back-splicing events. RNA editing, performed by adenosine deaminases acting on RNA (ADARs), also plays a role in circRNA formation, as A-to-I RNA editing can decrease the efficiency of intron pairing and back splicing (Shen et al. 2022). circRNA can be classified on their mode of formation into exonic circRNAs (ecircRNAs), originating from one or more exons of the pre-mRNA (Meng et al. 2016), intronic circRNAs (ciRNAs), and exon-intron circRNAs, that contains both introns and exons (EIciRNAs) (Fig. 1) (Guo et al. 2014; Li et al. 2015). Even if in general, the back-splicing event is much less common than the canonical splicing, in specific biological condition, circRNAs can be even more abundant than their linear counterparts (Qu et al. 2017). circRNAs are synthesized in the nucleus, but they predominately localize in the cytoplasm (Kristensen et al. 2019).

**Fig. 2:**
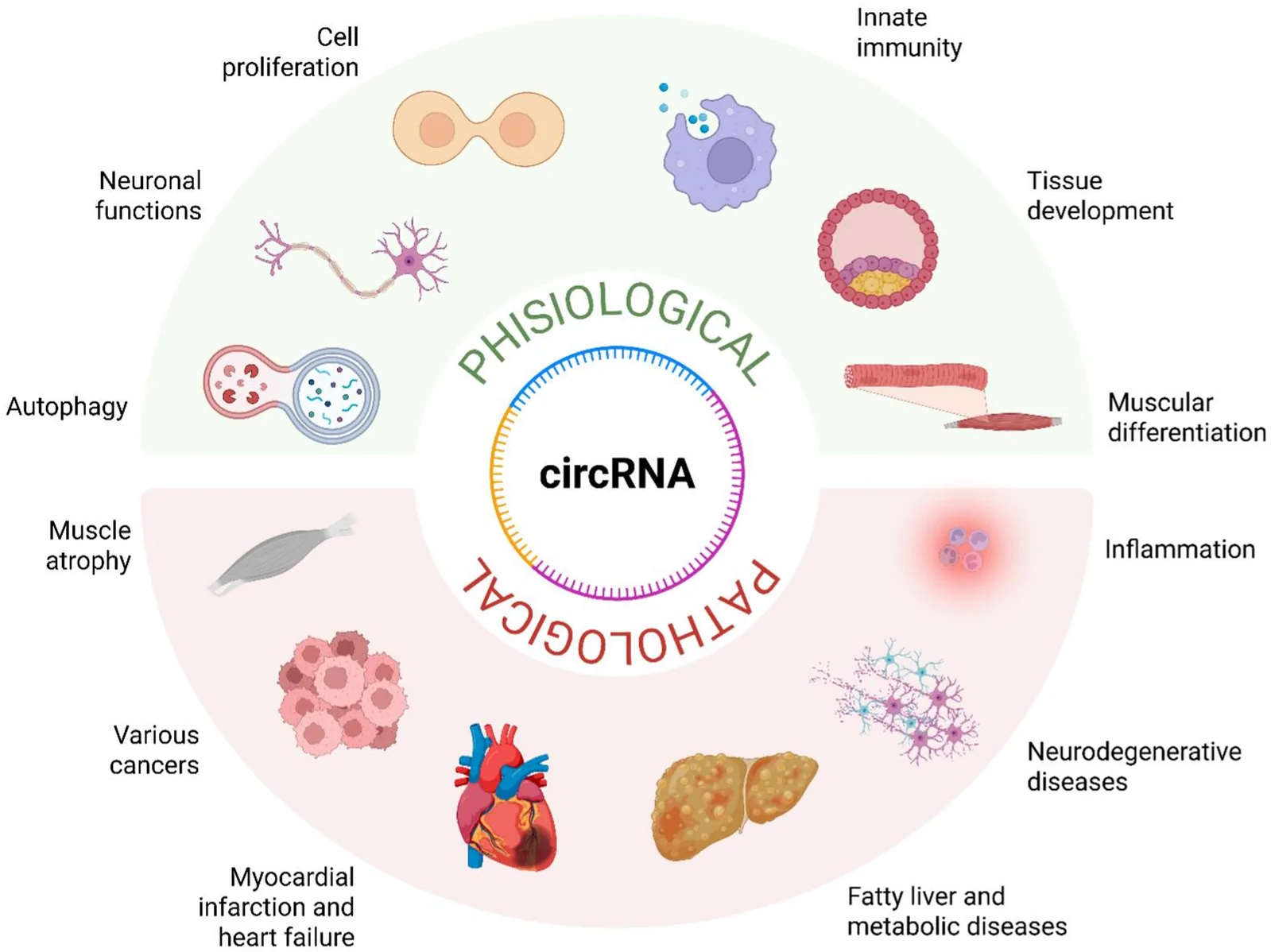
Physiological and pathological functions of circRNAs in different organs and tissues

circRNAs possess roles and functions that are distinct and independent from those of their linear counterparts. Indeed, circRNA act through a variety of mechanisms including miRNA sponging, interaction with RNA binding proteins, regulation of transcriptional and post-transcriptional gene expression. Recent findings have demonstrated that they can be also translate into proteins (Fig. 1). To cite only a few of the key biological processes in which circRNAs play critical roles, circRNA are involved at several stages of development, in the regulation of cellular proliferation, in the innate immunity pathways, in autophagy and in neuronal functions (Misir et al. 2022). According to their key role in those processes, dysregulation of expression levels of specific circRNA has been associated to various cancer, muscle diseases, metabolic diseases, inflammation and neurodegenerative disorders (Fig. 2) (Dube et al. 2019; Fischer and Leung 2017; Mai et al. 2022).

## circRNA in neuronal tissue

circRNAs are specifically abundant in the neuronal tissues, in part due to their generally exceptional stability and to the enrichment and importance of alternative splicing mechanisms in this tissue (Dong et al. 2023; Kirio et al. 2025; Weyn-Vanhentenryck et al. 2018). In the nervous system, circRNAs display highly cell‑type‑specific, developmental stage‑specific, and region‑specific expression patterns, reflecting their involvement in the regulation of neuronal identity and function. circRNAs participates in those mechanisms, with all their mode of action, i.e. as molecular sponges for microRNAs, interacting with RNA‑binding proteins, or being translated into micropeptides (Li et al. 2021).

Regulation of circRNA nuclear export is another crucial aspect of their biology in the nervous system, as recent findings indicate that the nuclear-cytoplasm transport can be important in regulating circRNA function in neuronal tissues. Indeed, it has been demonstrated that circRNAs enriched in (A)-rich motifs rely on differentiation‑dependent export pathways during neuronal development. These specialized export routes contribute to the dynamic redistribution of circRNAs between nuclear and cytoplasmic compartments throughout maturation and synaptic formation (D. Li and Huang 2024).

Interestingly, circRNAs abundance increases not only during neuronal differentiation but also in the aging brain (Kirio et al. 2025; Westholm et al. 2014). Dysregulation of circRNA expression has been implicated in a wide range of neuropathological conditions, including neurodegenerative diseases such as Alzheimer’s disease, Parkinson’s disease, and frontotemporal dementia, as well as psychiatric disorders including schizophrenia and bipolar disorder (for a review see (Li et al. 2021). Aberrant circRNA expression observed in patient tissues or in cellular or animal model systems suggest that circRNAs may contribute to neurodegenerative disease onset. circRNA alteration has been described in altered synaptic function, neuroinflammation, and in protein aggregation pathways (Huang et al. 2025). Additionally, circRNAs are increasingly recognized as key players in neural injury and regeneration. Specific circRNAs modulate responses to peripheral nerve injury, traumatic brain injury, and spinal cord damage by influencing processes such as axonal regrowth, immune activation, and glial cell responses (Li and Huang 2024; Zong et al. 2024). In conclusion, the present view highlights circRNAs as key players in many aspects of neuronal life and suggest their potential future use as therapeutic targets for neural repair strategies.

## circRNAs in muscle tissues

Even if circRNAs are particular abundant in the brain, they are also widely expressed in other tissues, including skeletal muscle (Rybak-Wolf et al. 2015; Sun et al. 2022). Consistent with this distribution, an increasing number of studies have reported alteration of circRNA expression and localization not only in neurodegenerative diseases (Ziaei et al. 2026) but also in muscle disease (Ramadan et al. 2022; Urzi et al. 2025). Myogenesis is a complex phenomenon finely regulated by the interplay of transcription factors and different species of noncoding RNA, which together ensure the proper progression of muscle formation (Andresini et al. 2019; Ballarino et al. 2016; Maione 2018). Within the complex regulatory network that governs muscle formation, circRNAs have recently emerged as additional contributors to the control of skeletal muscle growth and differentiation, adding a new layer of regulation alongside the well-known roles of lncRNAs and miRNAs (Greco et al. 2018). Early high-throughput sequencing analyses revealed that thousands of circRNAs change dynamically during myogenic differentiation in both humans and mice, with several hundred showing conservation across species (Legnini et al. 2017). Successive functional studies, including broad siRNA-based screens, demonstrated that a number of these circular transcripts have direct and measurable effects on muscle development. Among the first functionally investigated examples, circBNC2 is generated from the BNC2 gene, which encodes a zinc-finger transcriptional regulator. During myogenic differentiation, circBNC2 strongly accumulates while the corresponding linear mRNA decreases. Although circBNC2-specific depletion did not directly affect differentiation, these opposite expression profiles suggest that circularization may reduce the production of the anti-myogenic BNC2 transcript when differentiation begins (Legnini et al. 2017). Another relevant example is circZNF609, which derives from the ZNF609 gene and is altered in both rhabdomyosarcoma and Duchenne muscular dystrophy (Rossi et al. 2019; Wang et al. 2019). Selective depletion experiments demonstrated that circZNF609 supports myoblast proliferation, independently of its linear counterpart, and reduces myotube formation partly through interaction with miR-194-5p. Moreover, circZNF609 contains a circular RNA-specific open reading frame that can be translated (Legnini et al. 2017; Wang et al. 2019). Although both circRNAs originate from genes encoding zinc-finger regulatory proteins, they are discussed here because of their experimentally established roles in muscle biology rather than this shared feature of their host genes. Finally, in goat embryonic muscle, the well-characterized circRNA CDR1as can be transcriptionally activated by MYOD. In this conditions, CDR1as acts as a competing endogenous RNA (ceRNA) for its primary miRNA partner, miR-7, thus participating in the regulation of IGF1R mRNA and enhancing satellite cell differentiation (Li et al. 2019).

## Polyamines, SMOX and circSMOX

**Fig. 3:**
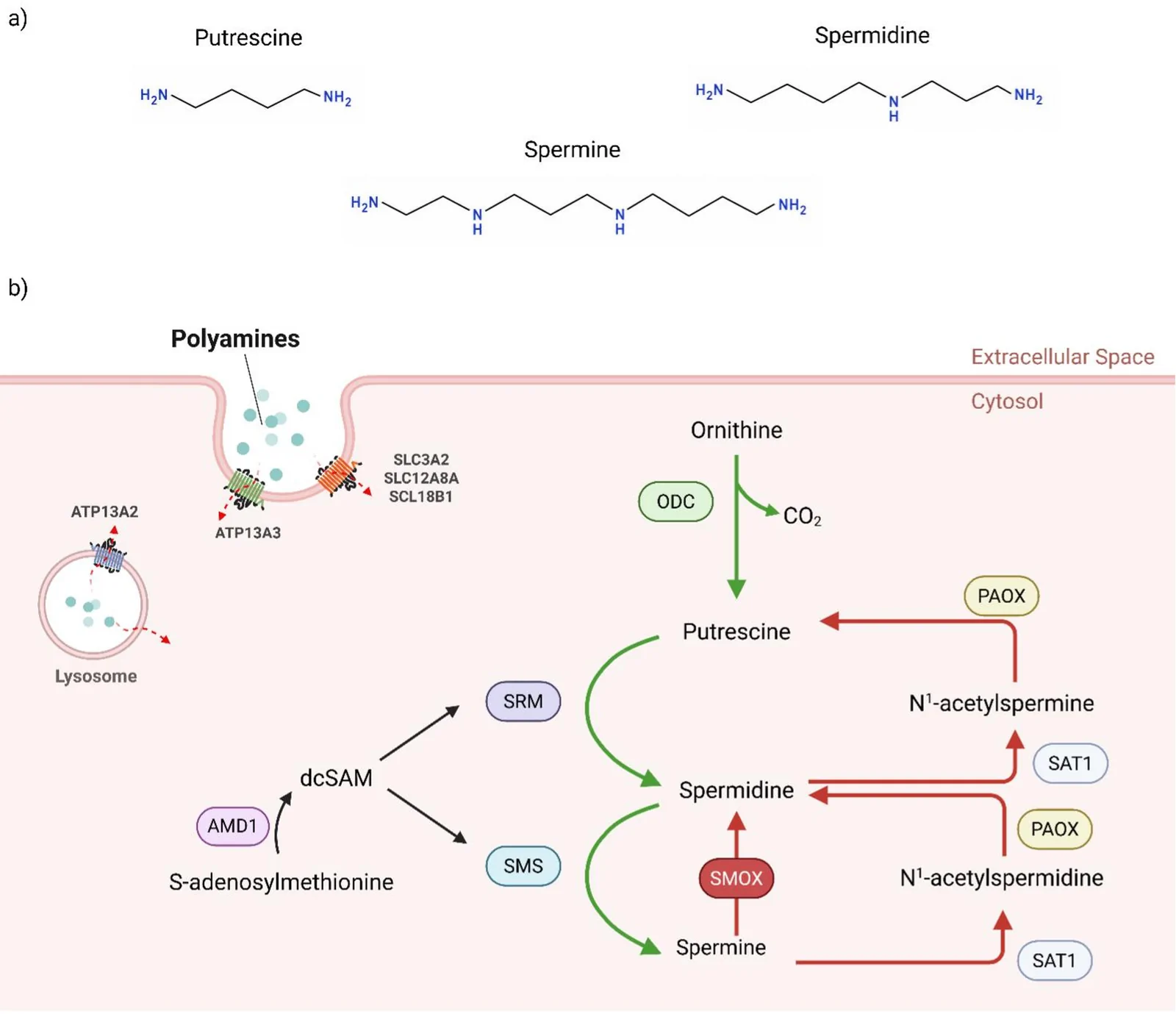
Polyamines and polyamine metabolism.** a** The structure of the main polyamines putrescine, spermidine and spermine are reported. **b** Schematic view of the cellular polyamine metabolism: Polyamine transporters are shown on the cell membrane and in the lysosome: ATP13A3, ATP13A2, solute carrier (SLC) SLC3A2, SLC12A8A, and SLC18B1. The biosynthetic and the catabolic route are depicted in green and red, respectively. AMD1, Adenosylmethionine Decarboxylase 1; dcSAM, decarboxylated S-adenosylmethionine; SRM, spermidine synthase; SMS, spermine synthase; ODC, ornithine decarboxylase; PAOX, polyamine oxidase; SAT1, spermidine/spermine N1-acetyltransferase 1; SMOX, spermine oxidase

Polyamines, namely putrescine, spermidine and spermine (Fig. 3A), are small molecules that exert an incredible wide range of functions within the cells, spanning from the interaction with nucleic acid to regulation of translation and ion channel permeability. Polyamine levels within the cells are strictly regulated by balancing biosynthesis, catabolism and transport (Fig. 3B). In particular, biosynthesis proceeds from ornithine to putrescine thanks to the enzyme ornithine decarboxylase (ODC1), then spermidine and spermine are synthesized by the activity of spermidine synthase (SRM) and spermine synthase (SMS) respectively. Catabolism of polyamines involves the acetylation od spermine and spermidine by the spermidine/spermine N1-acetyltransferase 1 (SAT1) and the subsequent oxidation by the enzyme polyamine oxidase (PAOX). A parallel catabolic enzyme, spermine oxidase (SMOX), oxidases spermine to give spermidine. Increasing evidence has linked alterations in the expression and activity of enzymes regulating polyamine metabolism to the onset and progression of diverse pathological conditions, including cancer, inflammatory disorders, and neurodegenerative diseases (Casero et al. 2018; Casero and Pegg 2009; Cervelli et al. 2014). In particular, alterations of SMOX expression have been linked to brain and muscle diseases. SMOX expression results increased in rat brain after cerebral artery occlusion (Fan et al. 2019) and in mice subjected to traumatic brain injury. In neocortex, SMOX overexpression produces age-dependent neuronal loss, glial activation and markedly enhanced vulnerability to kainate-induced excitotoxic injury (Cervetto et al. 2021). Moreover, at a molecular level, SMOX-overexpressing brains display increased oxidative DNA damage (Leonetti et al. 2020) nuclear Nrf2, altered phosphorylation of glutamate-receptor subunits, reduced expression of excitatory amino-acid transporters and increased astrocytic system x_c⁻ activity, with impaired glutamate uptake and abnormal extracellular glutamate handling, strongly linking SMOX activation to excitotoxicity (Cervelli et al. 2022; Pietropaoli et al. 2018). Pharmacological SMOX inhibition mitigates neuroinflammation and neuronal degeneration (Fan et al. 2019) and SMOX silencing preserves blood–brain barrier integrity, reducing endothelial oxidative and inflammatory injury (Wang et al. 2024). Transcriptomic data from post-mortem tissue showed an age-related increase in human brain SMOX expression (J.-M. Sun et al. 2025).

In skeletal muscle, by contrast, SMOX activity appears to be trophic and differentiation-associated, increasing during C2C12 myogenesis (Cervelli et al. 2009). SMOX expression is decreased in adult mouse muscle by immobilisation, fasting, denervation and ageing (Bongers et al. 2015). Moreover, a decrease in SMOX expression has been found in gastrocnemius of ALS mouse models (Reinoso-Sánchez et al. 2020).

**Fig. 4:**
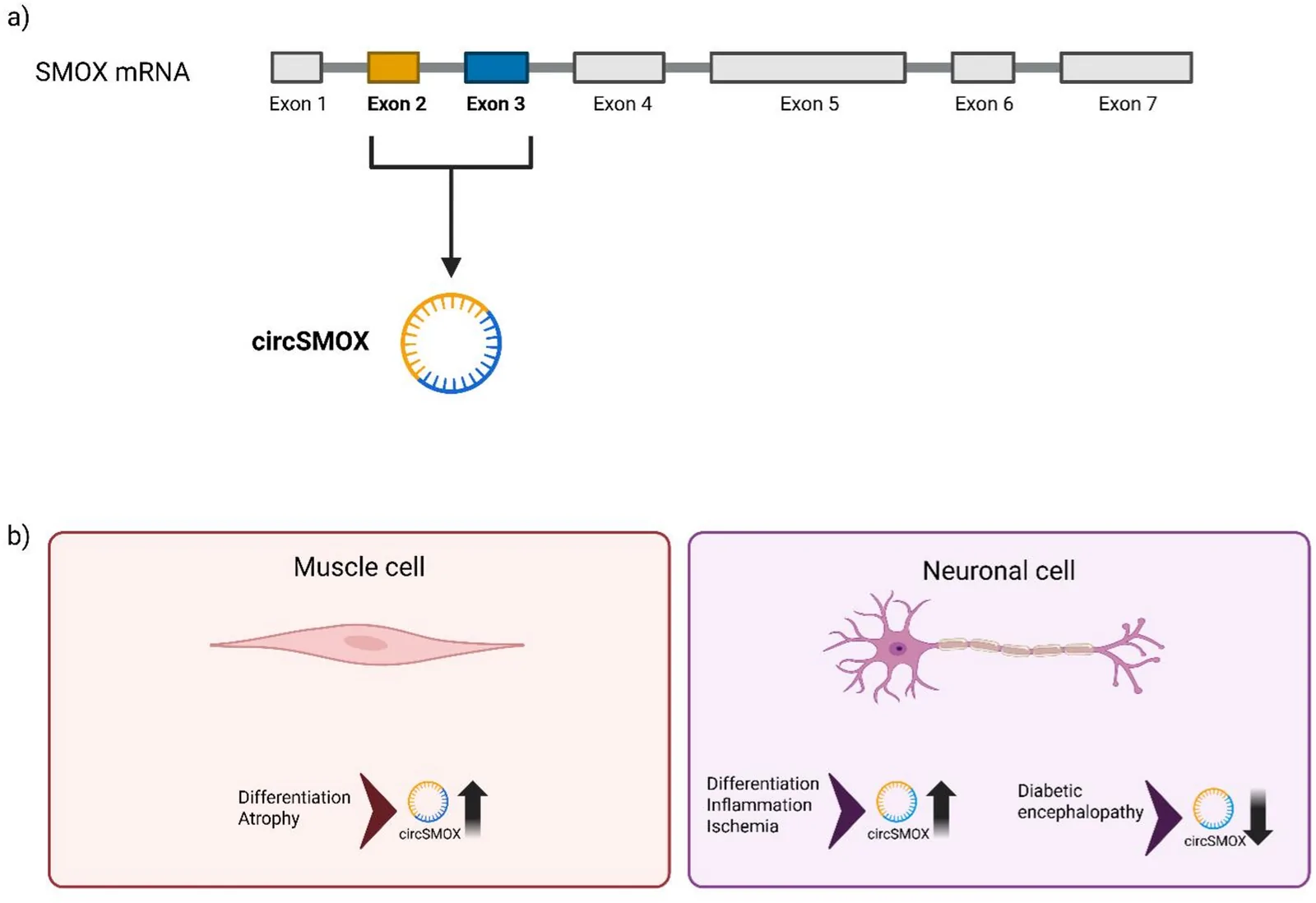
circSMOX structure and functions.** a** circSMOX arises from exons 2 and 3 of SMOX transcript. **b** circSMOX functions in muscle and neuronal cells

These findings strongly demonstrate that SMOX expression is deregulated in different pathological conditions and suggest a complex networks of regulations. To better depict this complexity, it is important to mention that murine SMOX gene is able to give rise to at least 9 splicing variants (Cervelli et al. 2004) and to a circRNA, named circSMOX. Unfortunately, so far, a human counterpart of the murine circSMOX has not been described. circSMOX (also known as mmu_circ_0001068 (circBase ID) (Rybak-Wolf et al. 2015; Weng et al. 2018) or circ_000595 (Mehta et al. 2017; Memczak et al. 2013) is a circular RNA approximately 460 nucleotides long derived from the murine SMOX gene. It is generated through a back-splicing event that covalently links exons 2 and 3 of the linear *SMOX* transcript, as illustrated in Fig. 4A and previously reported (Reinoso-Sánchez et al. 2020). Subcellular localization studies in skeletal muscle–derived cell lines indicate that circSMOX is mainly localized in the cytoplasm, consistent with a potential role in post-transcriptional regulation. Notably, the abundance of circSMOX is considerably lower than that of the canonical linear SMOX mRNA, with expression levels typically one to two orders of magnitude lower (Reinoso-Sánchez et al. 2020). To date, circSMOX has been detected in both neuronal (Han et al. 2023; Liu et al. 2022; Mehta et al. 2017) and skeletal muscle (Reinoso-Sánchez et al. 2020; Wang et al. 2019) tissues. The first studies identified back-splicing specific reads in RNAseq analysis (Mehta et al. 2017; Memczak et al. 2013; Rybak-Wolf et al. 2015; Weng et al. 2018) and only more recently an experimental validation confirmed the presence of circSMOX in both tissues (Han et al. 2023; Liu et al. 2022; Reinoso-Sánchez et al. 2020). In the following paragraphs, we summarize the findings reported so far on circSMOX and discuss its emerging biological relevance.

## circSMOX in neuronal tissues

As reported above, circRNAs are particular abundant in neuronal tissues where they contribute to brain development and affect the aetiology of neurologic diseases (Mehta et al. 2020). circSMOX was firstly annotated in mouse brain and ES cells samples by Memczak and co-workers (Memczak et al. 2013). Some years later, the data was confirmed by an independent study (Rybak-Wolf et al. 2015). In that study, an extensive analysis of 29 RNA-sequencing datasets from both human and mouse brain detected circSMOX in synaptosomal fractions as well as in the cytoplasm of mouse brain samples. Furthermore, the analysis of in vitro models (P19 cell differentiation and primary neuron maturation) revealed the detection of the backsplicing reads of circSMOX. Notably, the expression of circSMOX was detected throughout primary neuron differentiation, from day 0 to day 28, with a pick at day 14 supporting a potential role during brain development.

Altered circSMOX expression has also been reported under pathological conditions. Indeed, the profiling of circRNAs after transient focal ischemia indicated an induction of circSMOX at early times (6 h) after reperfusion (Mehta et al. 2017).

In both in vitro and in vivo models of diabetic encephalopathy, a pathological complication of diabetes mellitus (Nagayach et al. 2024), circSMOX has been identified as one of the 183 altered circRNA (Liu et al. 2022). In particular, its expression is downregulated both in the hippocampus of diabetic mice and in cultured neurons treated to mimic diabetic encephalopathy (Liu et al. 2022). Additionally, undifferentiated PC12 cells treated with Lipopolysaccharide (LPS) has been used as an in vitro model of spinal cord injury, and in these conditions circSMOX has been found to be upregulated after LPS stimulation. Overexpression of circSMOX increase apoptosis and cytokines production under inflammatory conditions. Importantly, circSMOX was shown to bind miR-340‐5p, functioning as a miRNA sponge. Since miR‐340‐5p targets the protein SMURF1, an E3-ubiquitin ligase implicated in neuroinflammation (Shao et al. 2018), these findings suggest that circSMOX could play a role in neuroinflammation through the miR‐340‐5p/SMURF1 axis (Han et al. 2023).

## circSMOX in muscle tissues

circSMOX has been identified also in muscle tissues. In a comprehensive analysis of circRNA expression during myogenic differentiation, circSMOX reads were identified in differentiated muscle cells (Legnini et al. 2017). During this process, circSMOX as well as its linear counterpart display a similar bell-shaped expression profile, increasing during early differentiation and subsequently declining. However, under atrophic condition, their regulation diverges: linear SMOX expression decreases while circSMOX expression increases (Reinoso-Sánchez et al. 2020). A difference between the expression patterns of a circular RNA and its corresponding linear transcript often indicates that the circular form may carry out regulatory activities that are independent of the canonical mRNA. Skeletal muscle atrophy is characterized by a progressive loss of muscle mass and strength and may result from a wide range of conditions, including prolonged inactivity, inadequate nutrition, severe burns, aging, and numerous chronic illnesses such as heart failure, chronic renal dysfunction, AIDS, sepsis, immune‑related disorders, cancer, and various muscular dystrophies (Fanzani et al. 2012). In the gastrocnemius muscle of two amyotrophic lateral sclerosis (ALS) mouse models, circSMOX expression is increased compared with wild type animals and with corresponding linear transcript. Time course analyses showed that circSMOX expression accumulates during disease progression, reaching maximal levels at the end-stage disease, suggesting its possible use as a biomarker of disease evolution (Reinoso-Sánchez et al. 2020). Consistently, transcriptomic profiling following peripheral nerve injury also reported increased circSMOX expression in atrophic muscle (Weng et al. 2018).

## Conclusion and future perspective

In conclusion, the expression of circSMOX appears to be present in muscle and neuronal samples and to be induced under specific physiological and pathological conditions (Fig. 4B). Despite the available evidence, much remains to be elucidated regarding the role and function of circSMOX. Its molecular mechanism of action is still largely unclear, although some evidence suggests a potential role as a miRNA sponge. Nevertheless, additional modes of action need to be investigated, including potential interactions with long non-coding RNAs or other RNA-binding partners, as well as the possibility that it may be translated into a functional protein product. It would be interesting to investigate if circSMOX can regulate the expression of its linear counterpart, thus participating in the control of polyamine homeostasis. A recent study identified a circRNA of 1361 nucleotides arising from human *ODC1* gene, circODC1 (Jin et al. 2024). Interestingly, this circRNA acts in a ceRNA network with ODC1 mRNA, sponging the miR-607 and thus increasing stability of ODC1 mRNA. To our knowledge, this is the first evidence of a circRNA arising from polyamine related genes that can regulate polyamine metabolism. A recent analysis in circBase database (http://www.circbase.org/) identified several different circRNAs potentially arising from other polyamine related genes, in particular from the ODC1 regulators *OAZ1*,* OAZ2*,* AZIN1 and AZIN2* genes (M. N. Rossi et al. 2024) and even if the existence of those circRNAs needs to be experimentally validated, it is tempting to speculate that many other circRNA might participate in the polyamine homeostasis. Finally, another important avenue for future research is the identification of the human circSmox ortholog, which has not yet been described. The discovery of a conserved human ortholog would significantly strengthen the translational relevance of this molecule and could pave the way for new therapeutic perspectives both in neurodegeneration and in muscle atrophy.

## Data Availability

No datasets were generated or analysed during the current study.
